# GWAS and drug targets

**DOI:** 10.1186/1471-2164-15-S4-S5

**Published:** 2014-05-20

**Authors:** Chen Cao, John Moult

**Affiliations:** 1 Institute for Bioscience and Biotechnology Research University of Maryland 9600 Gudelsky Drive, Rockville, MD 20850, USA; 2 Computational Biology, Bioinformatics, and Genomics, Biological Sciences Graduate Program University of Maryland, College Park, MD 20742, USA; 3 Department of Cell Biology and Molecular Genetics University of Maryland, College Park, MD 20742, USA

## Abstract

**Background:**

Genome wide association studies (GWAS) have revealed a large number of links between genome variation and complex disease. Among other benefits, it is expected that these insights will lead to new therapeutic strategies, particularly the identification of new drug targets. In this paper, we evaluate the power of GWAS studies to find drug targets by examining how many existing drug targets have been directly 'rediscovered' by this technique, and the extent to which GWAS results may be leveraged by network information to discover known and new drug targets.

**Results:**

We find that only a very small fraction of drug targets are directly detected in the relevant GWAS studies. We investigate two possible explanations for this observation. First, we find evidence of negative selection acting on drug target genes as a consequence of strong coupling with the disease phenotype, so reducing the incidence of SNPs linked to the disease. Second, we find that GWAS genes are substantially longer on average than drug targets and than all genes, suggesting there is a length related bias in GWAS results. In spite of the low direct relationship between drug targets and GWAS reported genes, we found these two sets of genes are closely coupled in the human protein network. As a consequence, machine-learning methods are able to recover known drug targets based on network context and the set of GWAS reported genes for the same disease. We show the approach is potentially useful for identifying drug repurposing opportunities.

**Conclusions:**

Although GWA studies do not directly identify most existing drug targets, there are several reasons to expect that new targets will nevertheless be discovered using these data. Initial results on drug repurposing studies using network analysis are encouraging and suggest directions for future development.

## Introduction

Until recently, information on which variants within the human genome contribute to increased risk of common human disease was fragmentary and often statistically weak. New chip-based technologies and large-scale sequencing have now provided relatively unbiased and reliable information on SNVs (single nucleotide variants) and indels that are significantly associated with altered risk for a number of common diseases. To date, most information has been obtained through genome wide association studies (GWAS) using microarray technology, providing information only on common SNVs (the single nucleotide polymorphisms, SNPs). The current generation of GWA studies typically include several thousand individuals with the disease of interest and a similar number of control individuals without the disease. These studies and meta-analyses combining data from multiple studies have now found more than 1600 loci where variants are associated with complex traits, including many diseases (the GWAS catalog, http://www.genome.gov/gwastudies).

There have been a number of discussions on the efficacy of GWA studies [1]. In spite of the success in discovering disease associations, it is becoming clear that many disease mechanism genes with the highest effect on disease phenotypes are not discovered by GWAS. Studies of blood pressure provide a striking example. There is a long history of identification of genes affecting blood pressure using non-genomic methods, and 30 genes discovered in this way have provided successful targets for treating hypertension [2]. But only a few of these candidate genes and no drug targets are discovered in large scale GWAS [3]. Further, mouse knockout data suggest that some of the missing genes have very large effect sizes, with blood pressure changes of 10s of mm of Hg [4], whereas the largest changes associated with marker SNPs in GWAS studies are between about 0.5 and 1 mm of Hg.

Known drug targets - genes that usually have a large effect size on the corresponding disease phenotype, and so should be found by GWAS - provide a means of investigating whether non-discovery of mechanism genes is a general phenomenon. Here, we compare a set of reported mechanism genes in the GWAS catalog (http://www.genome.gov/gwastudies[5], January 2012) with a corresponding set of known drug target genes (obtained from Drugbank [6], January 2012) for the same diseases. We find that the overlap of these two sets is very low. We also investigate two possible explanations for low overlap. Finally, we consider the relationship between GWAS genes and drug targets in the context of a protein functional interaction network, and develop a machine learning method to predict new drug targets using the relationship between GWAS genes and known drug targets.

## Results

### Comparison of the GWAS catalog and Drugbank shows GWAS only detects a very small fraction of existing drug targets

We examined the relationship between genes in the GWAS catalog [5] and drug target genes in Drugbank [6]. The GWAS catalog (http://www.genome.gov/gwastudies/) is a comprehensive collection of results from published GWAS studies on a wide variety of disease and other traits such as height. Drugbank [6] is a database that combines detailed drug (i.e. chemical, pharmacological and pharmaceutical) data with comprehensive drug target information (sequence, structure, and pathway). We compiled a list of disease related traits in the GWAS catalog and extracted the reported genes for each of them. The disease list includes a number of cancers, a variety of complex trait diseases, and disease predisposition traits such as obesity and hypertension. We then found the drugs used in treatment of each of these traits in Drugbank, and extracted the drug target genes for each drug. Thus, for each trait, we have a list of GWAS reported genes and a list of drug targets. For the 88 GWAS diseases that have drugs in Drugbank, there are on average 29.2 GWAS reported genes and 24.0 drug targets for 19.9 drugs (Table 1). There are a total 23 instances of GWAS genes that are also drug targets for the same disease. Three of these genes are each drug targets for two different diseases, so that only 20 of the 856 drug target genes have been discovered in GWA studies of the corresponding traits. This is slightly larger than the overlap of approximately 5 from a completely random model, but is a very low number considering that altered activity of most drug target genes will influence the disease phenotype.

**Table 1:** Overlap between GWAS reported genes and drug targets

Disease	Number of Drugs	GWAS reported genes	Number of drug targets	GWAS overlap, same disease*	GWAS overlap, all diseases**
Acute lymphoblastic leukemia	6	19	10	0	3
Age-related macular degeneration	9	23	2	1	2
Allergic rhinitis	69	11	20	0	5
Alzheimer's disease	5	54	179	0	40
Amyotrophic lateral sclerosis	3	26	2	0	1
Ankylosing spondylitis	39	17	29	0	9
Arthritis	168	7	112	0	35
Asthma	102	43	52	1	19
Atopic dermatitis	12	8	3	0	1
Atrial fibrillation	45	7	25	0	14
Attention deficit hyperactivity disorder	3	81	1	0	1
Autism	3	6	10	0	5
Basal cell carcinoma	6	8	9	0	2
Bipolar disorder/Schizophrenia	93	215	110	1	32
Blood pressure/Hypertension	351	100	114	3	35
Breast cancer	84	42	43	1	13
Celiac disease	3	74	1	0	0
Chronic kidney disease	8	69	6	0	2
Chronic lymphocytic leukemia	14	17	29	0	5
Chronic myeloid leukemia	6	9	15	0	6
Chronic obstructive pulmonary disease	14	18	7	0	2
Colorectal cancer	8	14	16	0	6
Coronary heart disease	6	84	5	0	3
Crohn's disease	7	136	23	0	9
Cystic fibrosis	8	7	11	0	5
Depression/Depressive disorder	45	68	73	0	17
Diabetes	46	205	59	4	21
Duodenal ulcer	8	2	18	0	5
Emphysema	10	5	17	0	5
Endometrial cancer	1	2	2	0	0
Endometriosis	5	4	7	0	3
End-stage renal disease	2	2	8	0	3
Epilepsy	18	1	53	0	10
Esophageal cancer	1	18	2	0	1
Gallstones	1	1	1	0	0
Gastric cancer	2	3	1	0	0
Glaucoma	24	13	31	0	6
Glioblastoma	2	1	1	0	0
Heart failure	51	16	65	0	27
HIV/AIDS	54	62	53	1	9
Hodgkin's lymphoma	8	7	31	0	7
Hypertriglyceridemia	2	5	4	0	3
Hypothyroidism	5	43	8	1	5
Inflammatory bowel disease	2	18	8	0	4
Kawasaki disease	1	20	11	1	5
Malaria	17	3	17	0	4
Male infertility	6	5	3	0	3
Melanoma	9	20	6	0	0
Menopause age	9	23	15	0	4
Migraine	20	7	46	0	10
Multiple myeloma	7	3	10	0	3
Multiple sclerosis	10	123	30	1	12
Myocardial infarction	29	14	44	0	17
Narcolepsy	2	4	6	0	1
Nephropathy/Nephrotic syndrome	20	26	38	0	9
Neuroblastoma	2	2	6	0	2
Non-small cell lung cancer	5	7	10	0	1
Obesity	4	40	11	0	4
Osteoarthritis	26	3	46	0	10
Osteoporosis	13	10	10	0	2
Ovarian cancer	5	10	4	0	1
Paget's disease	4	9	6	0	1
Pancreatic cancer	2	29	11	0	4
Panic disorder	6	10	18	0	4
Parkinson's disease	20	62	184	1	34
Polycystic ovary syndrome	2	7	2	0	1
Prostate cancer	14	94	21	0	8
Psoriasis/Psoriatic arthritis	19	30	39	0	13
Refractive error	1	4	4	0	1
Restless legs syndrome	2	6	18	0	6
Rheumatoid arthritis	46	67	80	2	29
Sleepiness	1	2	2	0	0
Stevens-Johnson syndrome/toxic epidermal necrolysis	1	12	1	0	0
Stroke	8	4	7	0	6
Tardive dyskinesia	3	1	22	0	7
Testicular cancer	4	7	6	0	2
Thyroid cancer	2	5	3	0	2
Tuberculosis	12	5	18	0	4
Type 1 diabetes	8	74	18	0	8
Type 2 diabetes	28	91	34	3	13
Ulcerative colitis	5	95	9	1	6
Uterine fibroids	1	7	1	0	0
Venous thromboembolism	1	7	3	0	2
Vitiligo	4	25	8	1	2
Mean	19.90	29.18	24.00	0.26	7.09

*For each disease, the number of GWAS reported genes that are also drug targets for the disease.

**For each disease, the number of GWAS reported genes that are drug targets for any disease.

### Possible data related reasons for low overlap

One possible cause of lower overlap is that in Drugbank, some drug targets do not have a known mechanism and are probably 'predicted' targets based on sequence similarity to other verified drug targets [7,8], and thus may be incorrect. We therefore compiled a list of verified drug targets, all of which have known drug action mechanisms documented in Drugbank. We find similar results with this set to those for the complete list of drug targets. For those 353 drug targets for 81 diseases with known mechanisms and with corresponding GWAS studies, only 12 are discovered by GWAS (Additional file 1). On average, in this set there are 30 GWAS reported genes and 11.2 verified drug targets for each of these 81 diseases. A second possible cause of low overlap is mis-assignment of mechanism genes in the GWAS catalog. Marker SNPs (those associated with a trait) found in a GWAS locus are usually in linkage disequilibrium with many other SNPs covering a number of genes, any of which in principle might be in disease mechanism. In some cases, the catalog assignments may be incorrect, and the true mechanism gene in a locus may in fact be a drug target. We investigated the effect of this factor by comparing drug target/GWAS overlap described above with that obtained including all genes in each locus as candidates, rather than just those reported as candidates in the GWAS catalog. For the 58 diseases with sufficient information in the catalog, linkage disequilibrium expansion from marker SNPs increased the set of candidate genes from the 1997 reported to 4035, about a factor of two. The number of GWAS genes that are also drug targets increased from 18 to 24. This small increase is comparable with the increase of 3 that is expected from the random model. Thus, the number of GWAS/drug target matches missed as a consequence of misidentification of candidate genes appears very small. A third data related factor is coverage by the tag SNPs on the microarrays used in GWAS studies. If there is no tag SNP in linkage disequilibrium with the underlying variant involved in a disease mechanism, that contribution to the trait will not be detected. A study of 160 non-GWAS derived candidate genes for blood pressure concluded that only half were adequately covered with tag SNPs on a 500K array [3], suggesting this is a significant factor. But overall, data considerations do not qualitatively change the picture of very low GWAS gene/drug target overlap.

#### Analysis using 1000 genomes data shows Drug Target genes have fewer high frequency non-synonymous SNPs than GWAS reported genes

We next consider two possible reasons why GWAS identifies so few known drug targets. A study of all the SNPs in the GWAS catalog [5] has shown that reported SNPs are common (median risk allele frequency 36%, interquantile range (IQR) 21%-53%), and are associated with modest effect size (median odds ratio 1.33, IQR 1.20-1.61). We speculated that drug target genes may escape GWAS studies because these contain few common SNPs that affect function. To test this hypothesis, we examined the distribution of SNP frequencies and SNP effect size in GWAS identified genes and drug targets, using SNP frequencies calculated from 1000 genomes data [9].

A SNP may affect *in vivo *function of a gene product through a number of different mechanisms, including modified protein function or protein stability, altered regulation of gene expression, modified splicing, and changed stability of messenger RNA. We focus on non-synonymous SNPs, which have been shown to be significantly overrepresented at amongst GWAS marker SNPs [5]. We found that drug targets genes do have fewer non-synonymous SNPs (0.0155/residue vs. 0.0171/residue) and the tendency is more significant for common (Allele frequency > 5%) non-synonymous SNPs (0.00169/residue vs. 0.00221/residue, Mann-Whitney test P = 0.0017) (Table 2). We also included a set of predominantly monogenic disease genes from the Human Gene Mutation Database (HGMD) [10], expecting these to also be under negative selection pressure. SNP density is also lower in this class of genes. A possible explanation for the low occurrence of common SNPs is that the activity level of drug targets genes is strongly coupled to the disease phenotype. As a result they are under relatively high selection pressure, and SNPs with a substantial impact on function will be eliminated or tend to be at a low frequency.

**Table 2:** Comparison of common non-synonymous SNP densities between GWAS reported genes and drug targets

	Drug Targets	GWAS reported genes	HGMD genes	All genes
Density of all non-synonymous SNPs	0.0155	0.0171	0.0166	0.0171
Density of Common non-synonymous SNPs	0.00169 P = 0.0017^1^ P = 0.0023^2^	0.00221	0.00179	0.00214

^1^P-value for Mann-Whitney test against the density of common non-synonymous SNPs for GWAS reported genes.^2^P-value for Mann-Whitney test against the density of common non-synonymous SNPs for all genes.

#### Evolutionary analysis shows drug target genes are under slightly stronger negative selection than GWAS reported genes

If the drug targets genes are under stronger selection as we propose on the basis of SNP density, that effect should also be observable in the rate of sequence change during the evolutionary history of the gene family. The ratio of the rate of non-synonymous to synonymous change, dN/dS, [11] for a gene provides one measure to detect such selection pressure. We compared the dN/dS for GWAS and drug target genes using human-mouse and human-chimp data from H-invDB [12] and found both are under stronger selection (Table 3) than all genes. We found HGMD genes [10] also exhibit negative selection in recent history (dN/dS calculated using human-chimp orthologs). The selection against variants in drug target genes is slightly stronger than that against variants in GWAS reported genes (Table 3) for dN/dS calculated using human-chimp orthologs, suggesting the selection is stronger for drug targets in recent history.

**Table 3:** dN/dS analysis for GWAS reported genes and drug targets

		Number of genes	Mean dN/dS	P Value for Mann-Whitney test against all genes	P Value for Mann-Whitney test against GWAS reported genes
Human-Mouse orthologs	All genes	13691	0.22		
	GWAS reported genes	2932	0.19	2.44e^-09^*	
	Drug targets	1035	0.18	1.21e^-04^*	0.43
	Drug targets with known mechanism	432	0.17	6.04e^-06^*	0.038*
	HGMD genes	720	0.20	1.0	
Human-Chimpanzee orthologs	All genes	14173	0.44		
	GWAS reported genes	2911	0.36	1.26e^-13^*	
	Drug targets	1020	0.33	2.78e^-13^*	0.0098*
	Drug targets with known mechanism	423	0.32	4.20e^-08^*	0.013*
	HGMD genes	699	0.36	0.002*	

"*" denotes significant, i.e, P < 0.05

#### The influence of transcript length

**Figure 1:**
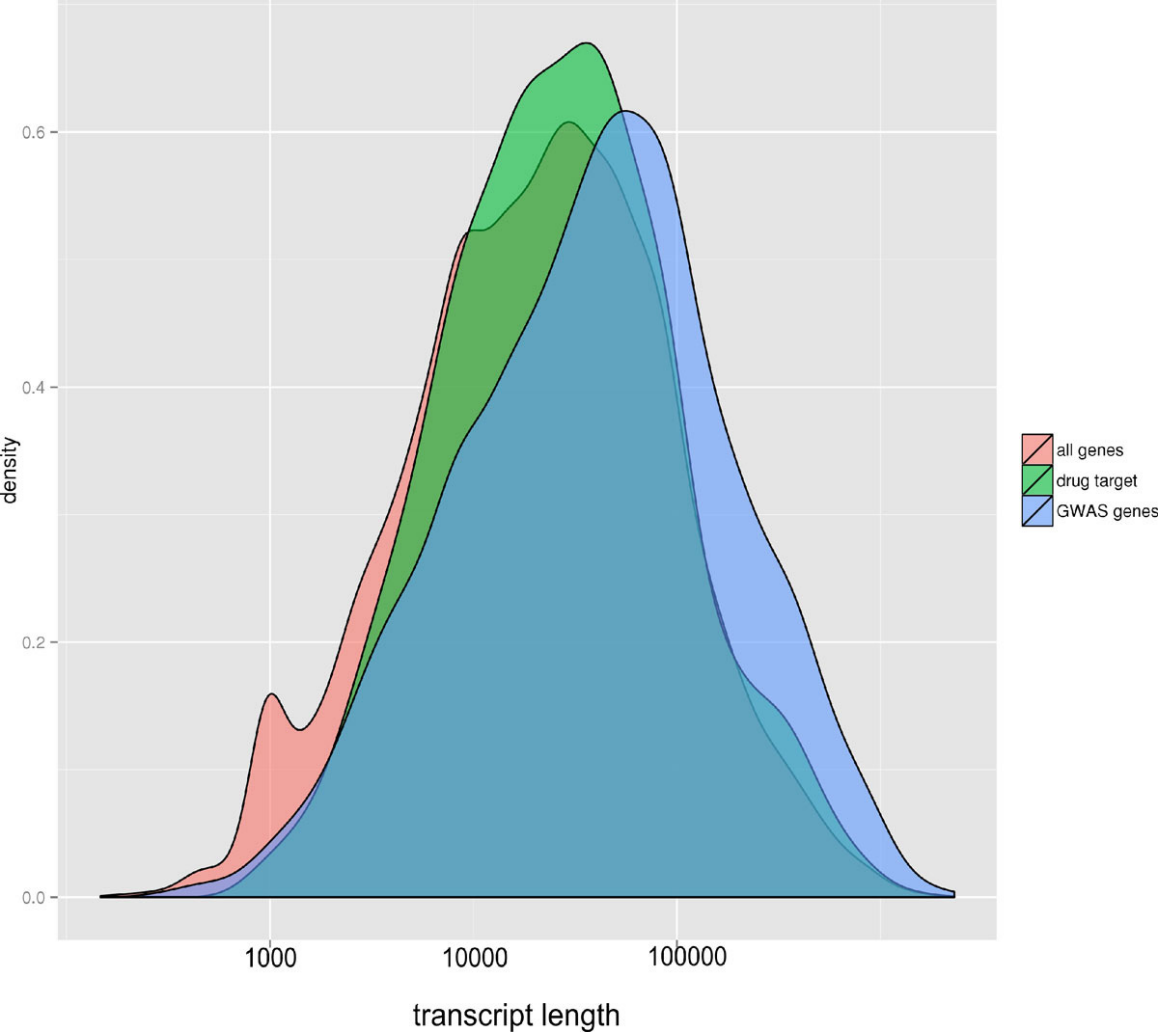
**Distribution of the log longest transcript length for different types of genes**. GWAS genes are on average substantially longer than drug target genes, and longer than the set of all genes.

For some mechanisms, for example those arising from missense SNPs, the probability of contributing to a complex trait is dependent on the length of the gene affected: Under similar selection pressures, the longer the gene, the more likely variants affecting gene function will be present. Other mechanisms, such as those directly affecting transcription rate, are not length dependent. To test for a length effect, we examined the length distribution for GWAS reported genes, for drug targets, and for all genes (Figure 1). GWAS reported genes are significantly longer than the drug target genes (paired Mann-Whitney test, P = 1.89e^-6^) and GWAS reported genes tend to be longer than all other genes. The mean longest transcript length for GWAS reported genes is about 110K while the mean longest transcript length for drug targets is about 60K, almost a factor two different. The outlier here is the GWAS gene set - drug targets have a similar distribution to that of all genes. Thus there is a strong length factor influencing whether or not variants in a gene contribute to a complex trait. This result is consistent with a role for length dependent mechanisms, although there could be other explanations.

#### Network analysis shows GWAS reported genes are close to drug target genes in a biological network

Although most drug targets are not identified through GWAS studies, they are obviously as much involved in the disease mechanism as GWAS genes, and so may be expected to have similar properties, particularly in terms of pathway and network relationships. A number of studies have incorporated network information to aid in identifying various classes of genes, for example using a network module formalism to combine signals from multiple GWAS studies [13,14] and using network flow models to predict drug targets from expression and other data in prostate cancer [15]. Network models have also been used to identify pathways implicated in cancer [16]. It has already been observed that GWAS genes are substantially more closely connected in a functional network [17] than random genes, and we expect that to be the case for other large effect genes, such as known drug targets.

There are many resources available for different types of human biological networks. Protein-Protein interaction data [18,19] have a wide coverage but usually have a high false positive rate. Curated pathways such as KEGG [20] and BioCarta (http://www.biocarta.com/genes/index.asp) are considered to be more accurate but the coverage is sparse [21]. For our purposes, networks built from other kinds of relationship, such as regulatory networks deduced from micro-array data [22,23] or networks based on biochemical reactions [24] are too narrow in terms of the interactions they capture.

In this study, we use the Functional Interaction (FI) network from [21], a protein functional interaction network generated by extending curated biological pathways with non-curated sources of information, including protein-protein interactions, gene co-expression, protein domain interaction, Gene Ontology (GO) annotations and text-mined protein interactions, and covering about 50% of human genes. The network strikes a balance between experimentally validated results and prediction, with the prediction portion benchmarked by a reasonably rigorous process. We were able to map 611 out of 821 drug targets genes and 1125 out of 1914 GWAS reported genes for the 88 diseases to the network.

**Figure 2:**
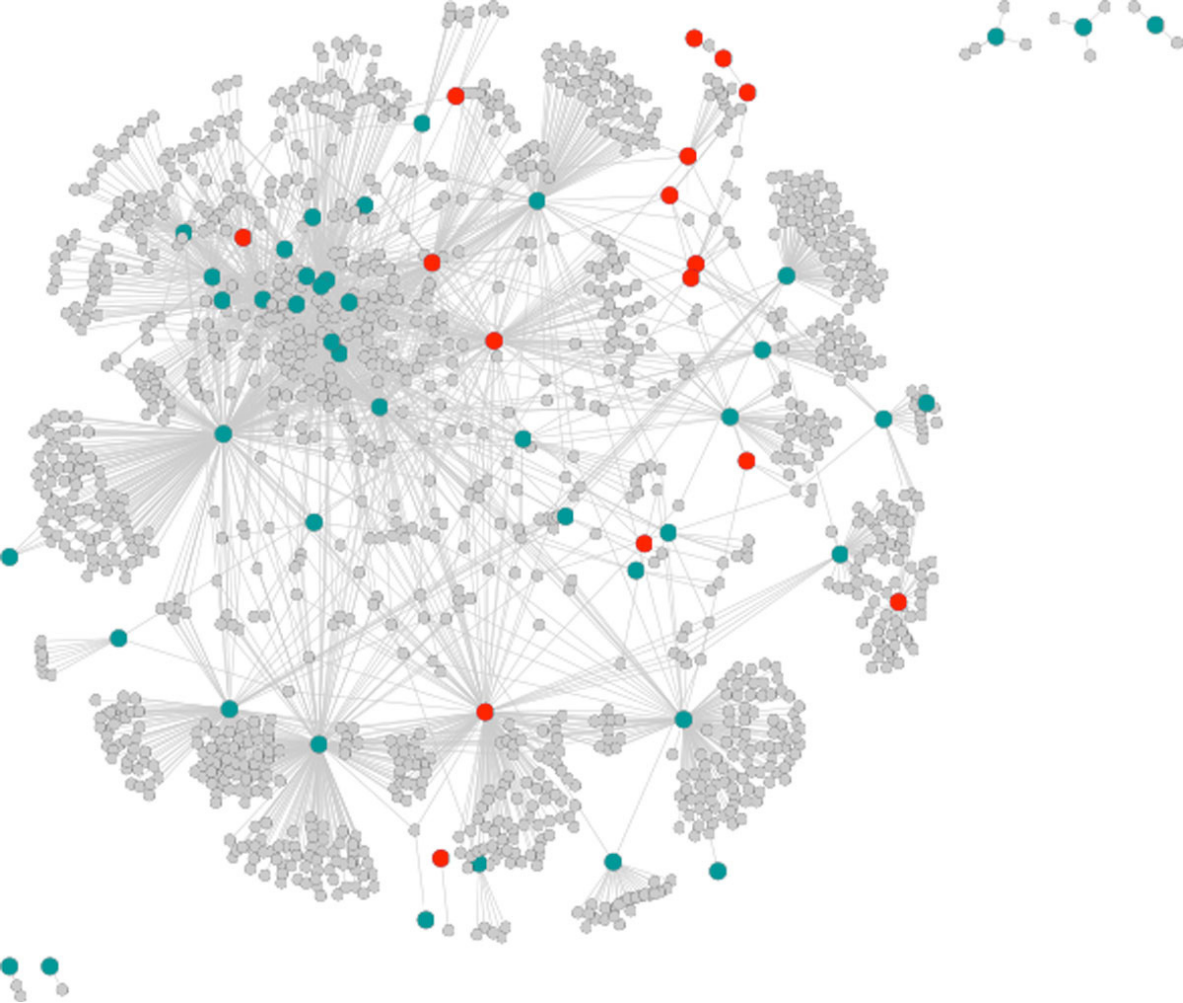
**Continuous network substructure formed by 43 of the 74 GWAS (green) and 16 of the 18 drug targets (red) for Type 1 Diabetes, allowing not more than one intermediate gene (grey)**. GWAS and drug target genes are intermingled in the network, and short paths are sufficient to form a connected network for almost all genes. FI network, figure from Cytoscape.

**Figure 3:**
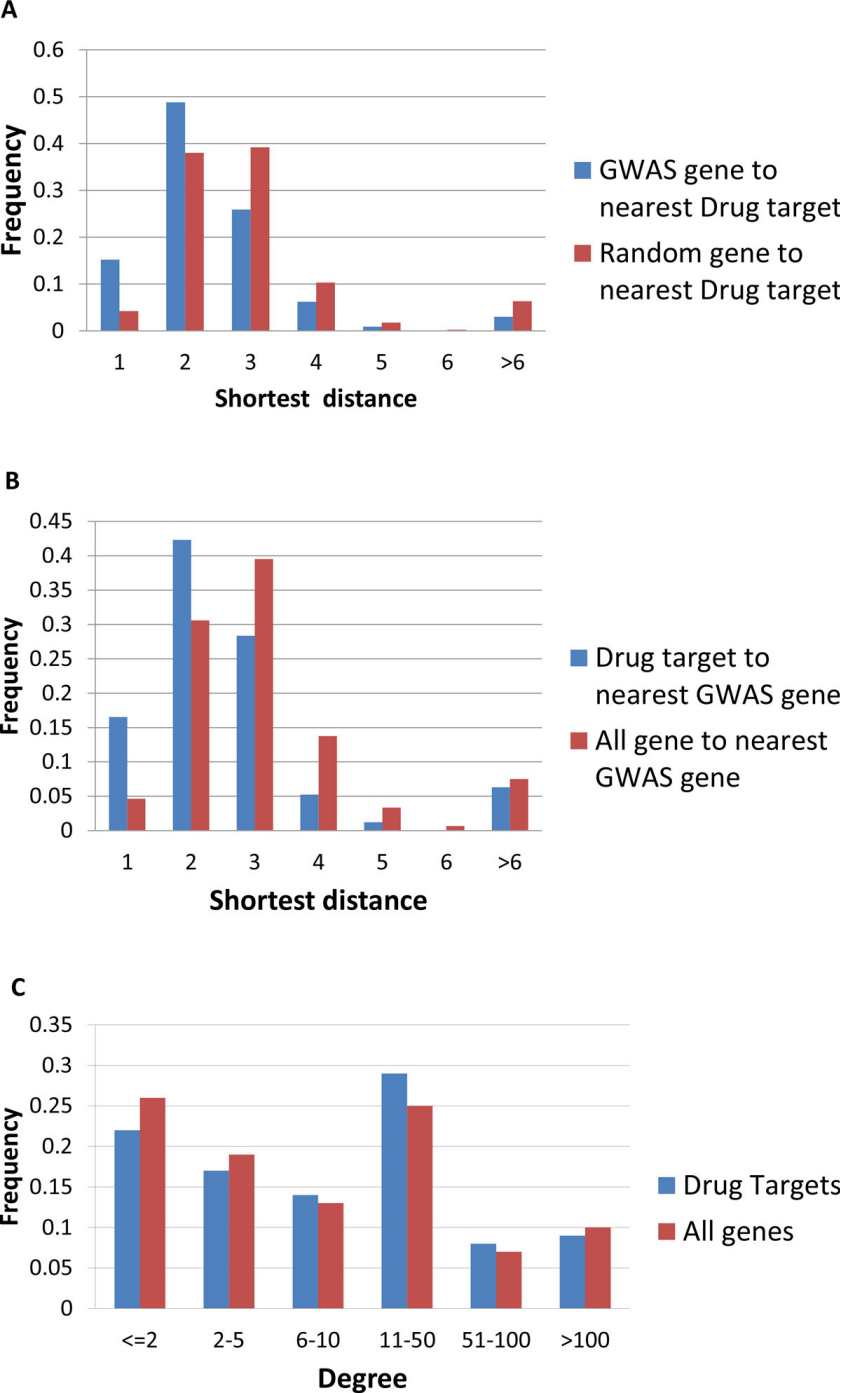
**A. Distribution of shortest distances to the nearest drug target for GWAS reported genes and all genes. B. Distribution of the shortest distance to the nearest GWAS genes for drug targets and all genes. C. Distribution of degree for drug targets and all genes in the FI network.** Drug targets have a slightly higher degree (Mann-Whitley test P = 0.014).

Examination of the network proximity of GWAS genes to each other and to drug targets for the same disease indeed shows a close-nit matrix of relationships. Figure 2 shows the network formed for the 43 GWAS and 16 drug target genes [6] for Type I Diabetes that project onto the FI network, and only including genes from these two sets which are linked by not more than one other intermediate gene. All drug targets and all but five of the GWAS genes form part of a single continuous sub-network. This suggests that the two sets of genes are indeed relatively close in their biological function. One measure of the relationship between GWAS reported genes and drug target genes is the closeness of each GWAS gene to its nearest drug target (Figure 3). The distributions show that distances from a GWAS reported gene to the closest drug target are on average much shorter than those of a random gene to a closest drug target, and the shortest distance from a drug target gene to the closest GWAS reported gene is also shorter than that of a random gene to the closest GWAS reported gene. Notably, drug targets are about three fold enriched in the first neighbors of GWAS genes and are also enriched in GWAS second neighbors (genes two steps away in the gene network) (Figure 3).

Highly connected genes have more neighbors, and thus are more likely to include GWAS genes as neighbors. Thus, the observed enrichment of short paths between drug targets and GWAS genes could partially be a consequence of higher connectivity for drug targets. To control for this effect, we compared the degrees of drug targets with all genes (Figure 3) and found drug targets have a slightly higher degree (Mann-Whitney test, P = 0.014) on average. However the difference is marginal, and is unlikely to significantly contribute to the substantial difference between the short path distribution for drug targets and all genes.

#### A machine learning method for drug target discovery

The relationship between drug targets and GWAS genes revealed in the network analysis suggests that it should be possible to identify potential new drug targets from GWAS genes using machine learning methods trained on network features. The idea is to evaluate the probability that any gene is a potential drug target, given its network environment. The environment of each gene is represented by a set of features. Since we observed a threefold enrichment of drug targets in the first neighbors of the GWAS genes, we use the number of GWAS neighbors for a gene as a feature. This quantity is highly dependent on the total number of neighbors a gene has, so we also use the degree of the gene as a control. As the previous analysis shows, second neighbors of drug targets genes (genes that are two steps away in the protein interaction network) are also enriched for GWAS genes, thus we also use the number of second neighbor GWAS genes of a gene as a feature. These three features capture the enrichment information from the analysis above, but there are some subtle relationships not included. The problem of identifying drug targets based on their relationship to GWAS genes is similar to the problem of finding missing relationships in social network analysis. We therefore also use common friends with GWAS genes, a widely used feature in the social network machine learning field [25]. The common neighbor feature is defined as the proportion of neighbors shared by two genes:

$$Common\;Neighbor\left(A,B\right)=\frac{count(N_{A}\cap N_{B})}{count(N_{A}\cup N_{B})}$$

In which N_A _is the set of Neighbors for gene A, N_B _is the set of Neighbors for gene B.

The total number of features for each gene is 3+N, where N is the number of GWAS genes for that disease that are mapped to the protein network. Since the number of drug targets (average 30) for a disease is very small compared to the total number of genes in the FI network (10956), the training set is highly unbalanced if we use the latter as the true negative set. To address this issue, we focus on the 932 existing drug targets in Drugbank that are also in the FI network, and thus restrict the task to identifying targets for existing drugs that can potentially be repurposed to treat other diseases. Repurposing is an attractive goal, since such use is much easier than developing a new drug from scratch [26].

We include the 30 diseases with at least 10 approved drug targets and 10 GWAS genes in the FI network. We tested four machine learning methods using the WEKA software package [27]: a SVM with a polynomial kernel, a SVM with a RBF kernel, a Naïve Bayes Network, and Random Forests. Among these the best result is achieved by a Random forest (Table 4). The best case is Kawasaki disease, with a true positive rate of 70% (recovering seven out of the 10 known drug targets) and a false positive rate of 2.7%.

**Table 4:** Machine learning results for different diseases, using a Random Forest.

Disease	GWAS genes	Drug targets (Mapped in Network)	True Positive	False Positive	Precision	Recall	ROC area	F-Measure
Ankylosing spondylitis	17	29(24)	0.36	0.123	0.074	0.36	0.73	0.123
Menopause	24	15(14)	0.571	0.098	0.082	0.571	0.819	0.143
Multiple sclerosis	126	30(28)	0.393	0.052	0.19	0.393	0.75	0.256
Myocardial infarction	14	44(40)	0.175	0.135	0.055	0.175	0.571	0.084
Nephropathy/Nephrotic syndrome	26	38(35)	0.371	0.245	0.056	0.371	0.576	0.097
Obesity	40	11(11)	0.273	0.098	0.032	0.273	0.724	0.058
Osteoporosis	10	10(10)	0.2	0.189	0.011	0.2	0.546	0.022
Pancreatic cancer	29	11(6)	0.167	0.1	0.011	0.167	0.611	0.02
Panic disorder	10	18(16)	0.438	0.118	0.061	0.438	0.754	0.107
Parkinson's disease	62	184(132)	0.606	0.226	0.307	0.606	0.712	0.407
Asthma	43	52(47)	0.213	0.102	0.1	0.213	0.713	0.136
Prostate cancer	95	21(18)	0.5	0.073	0.118	0.5	0.686	0.191
Psoriasis/Psoriatic arthritis	31	39(36)	0.5	0.076	0.209	0.5	0.852	0.295
Rheumatoid arthritis	67	80(68)	0.324	0.131	0.163	0.324	0.677	0.217
Type 1 diabetes	76	18(16)	0.25	0.104	0.04	0.25	0.631	0.07
Type 2 diabetes	92	34(28)	0.214	0.116	0.054	0.214	0.595	0.086
Bipolar disorder/Schizophrenia	217	110(81)	0.593	0.15	0.273	0.593	0.744	0.374
Blood pressure/Hypertension	101	114(102)	0.412	0.143	0.261	0.412	0.717	0.319
Breast cancer	43	43(38)	0.289	0.072	0.147	0.289	0.745	0.195
Chronic lymphocytic leukemia	17	29(26)	0.423	0.098	0.11	0.423	0.653	0.175
Colorectal cancer	14	16(16)	0.25	0.154	0.028	0.25	0.53	0.05
Acute lymphoblastic leukemia	19	10(10)	0.7	0.07	0.097	0.7	0.889	0.171
Crohn's disease	139	23(22)	0.455	0.093	0.105	0.455	0.764	0.171
Depression/Depressive disorder	68	73(62)	0.597	0.172	0.198	0.597	0.722	0.297
Diabetes	209	59(51)	0.216	0.081	0.134	0.216	0.712	0.165
Allergic rhinitis	11	20(19)	0.263	0.128	0.041	0.263	0.589	0.071
Glaucoma	14	31(25)	0.16	0.189	0.023	0.16	0.443	0.04
Alzheimer's disease	54	179(125)	0.544	0.178	0.321	0.544	0.69	0.404
Heart failure	16	65(54)	0.481	0.222	0.118	0.481	0.655	0.189
HIV/AIDS	63	53(34)	0.353	0.121	0.099	0.353	0.715	0.155
Kawasaki disease	20	11(10)	0.7	0.027	0.219	0.7	0.919	0.333

#### Potential new drug targets for drug repurposing

The 'false positive' drug targets are drug targets for other diseases which have very similar network properties to those of the disease under study. These may indeed be mistakes made by the classifier. However, some of these 'false positive' drug targets may be good candidates for repurposing, not previously identified.

For example, C1QB and C1QC are the highest scoring proteins in the false positive list for the best case, Kawasaki disease, These are subcomponents of complement C1Q. C1Q has been shown to be associated with lupus erythematous [28-30], another autoimmune disease related to Kawasaki disease [31,32], consistent with relevance to Kawasaki. C1Q is the target of several FDA approved drugs, for example, Etanercept, a drug treating rheumatoid arthritis and Adalimumab, a drug treating rheumatoid arthritis, psoriatic arthritis, ankylosing spondylitis, and other immune system mediated diseases. Thus these drugs may be potential candidates for use against Kawasaki disease.

Another disease where the method performs well is acute lymphoblastic leukemia (ALL), with a false positive rate of 7% and a true positive rate of 70%. There is a relatively long list of 'false positive' targets (Table 5). Careful inspection of these genes reveals some that may have relevance to acute lymphoblastic leukemia, and so drugs for which these are targets provide potential candidates for repurposing. For example, chromosomal aberrations (i.e. chromosome translocation) in FGFR1 are associated with stem cell myeloproliferative disorder and stem cell leukemia lymphoma syndrome (provided by RefSeq, Jul 2008). FGFR1 is the drug target of Palifermin, a recombinant human keratinocyte growth factor (KGF) for the treatment of oral mucositis associated with chemotherapy and radiation therapy. It's also the target for several experimental drugs.

**Table 5:** Top 'false positive' drug targets for acute lymphoblastic leukemia.

Target	Description from Refseq	Random Forest Probability
MAPK3	The protein encoded by this gene is a member of the MAP kinase family. MAP kinases, also known as extracellular signal-regulated kinases (ERKs), act in a signaling cascade that regulates various cellular processes such as proliferation, differentiation, and cell cycle progression in response to a variety of extracellular signals.	1
PIK3R1	Phosphatidylinositol 3-kinase plays an important role in the metabolic actions of insulin, and a mutation in this gene has been associated with insulin resistance.	0.96
RAF1	v-raf-1 murine leukemia viral oncogene homolog 1	0.96
EGFR	Mutations in this gene are associated with lung cancer. Multiple alternatively spliced transcript variants that encode different protein isoforms have been found for this gene	0.96
FGFR2	Mutations in this gene are associated with Crouzon syndrome, Pfeiffer syndrome, Craniosynostosis, Apert syndrome, Jackson-Weiss syndrome, Beare-Stevenson cutis gyrata syndrome, Saethre-Chotzen syndrome, and syndromic craniosynostosis.	0.96
KDR	This receptor, known as kinase insert domain receptor, is a type III receptor tyrosine kinase. Mutations of this gene are implicated in infantile capillary hemangiomas.	0.94
FLT1	This protein binds to VEGFR-A, VEGFR-B and placental growth factor and plays an important role in angiogenesis and vasculogenesis.	0.94
FGFR1	Chromosomal aberrations involving this gene are associated with stem cell myeloproliferative disorder and stem cell leukemia lymphoma syndrome.	0.94
IL2RG	The protein encoded by this gene is an important signaling component of many interleukin receptors	0.92
ERBB2	v-erb-b2 erythroblastic leukemia viral oncogene homolog 2, neuro/glioblastoma derived oncogene homolog	0.92
FGFR3	This particular family member binds acidic and basic fibroblast growth hormone and plays a role in bone development and maintenance. Mutations in this gene lead to craniosynostosis and multiple types of skeletal dysplasia.	0.9
AKT1	v-akt murine thymoma viral oncogene homolog 1	0.9
INSR	insulin receptor	0.9
IL2RA	Mutations in this gene are associated with interleukin 2 receptor alpha deficiency.	0.9
SDC2	The syndecan-2 protein functions as an integral membrane protein and participates in cell proliferation, cell migration and cell-matrix interactions via its receptor for extracellular matrix proteins. Altered syndecan-2 expression has been detected in several different tumor types.	0.88
MAPK1	The protein encoded by this gene is a member of the MAP kinase family. MAP kinases, also known as extracellular signal-regulated kinases (ERKs), act as an integration point for multiple biochemical signals, and are involved in a wide variety of cellular processes such as proliferation, differentiation, transcription regulation and development.	0.86
CD247	The protein encoded by this gene is T-cell receptor zeta, which together with T-cell receptor alpha/beta and gamma/delta heterodimers, and with CD3-gamma, -delta and -epsilon, forms the T-cell receptor-CD3 complex.	0.86
RET	ret proto-oncogene	0.86
VEGFA	vascular endothelial growth factor A	0.86
PTPN1	protein tyrosine phosphatase, non-receptor type 1	0.86
IL3RA	The protein encoded by this gene is an interleukin 3 specific subunit of a heterodimeric cytokine receptor.	0.84
HDAC1	histone deacetylase 1, Together with metastasis-associated protein-2, it deacetylates p53 and modulates its effect on cell growth and apoptosis.	0.82
CCND1	The protein encoded by this gene belongs to the highly conserved cyclin family, whose members are characterized by a dramatic periodicity in protein abundance throughout the cell cycle. This protein has been shown to interact with tumor suppressor protein Rb and the expression of this gene is regulated positively by Rb. Mutations, amplification and overexpression of this gene, which alters cell cycle progression, are observed frequently in a variety of tumors and may contribute to tumorigenesis	0.82
FASN	fatty acid synthase	0.82
CD4	The protein functions to initiate or augment the early phase of T-cell activation, and may function as an important mediator of indirect neuronal damage in infectious and immune-mediated diseases of the central nervous system.	0.8

A second potential repurposing target for acute lymphoblastic leukemia is the oncogene RET. Previous studies found differential expression of RET in acute myeloid leukemia [33], a distinct but related leukemia. In the version of Drugbank used in this analysis, there is no drug targeting RET for the treatment of ALL. Recently, however, the drug Ponatinib has been approved by the FDA for treatment of Philadelphia chromosome positive acute lymphoblastic leukemia (Ph+ALL) resistant or intolerant to prior tyrosine kinase inhibitor therapy. Thus, one of the high scoring ALL potential drug targets has now been approved for use for use with a new drug.

## Methods

### Connecting GWAS reported genes with drug targets using drug indication information from Drugbank

GWAS reported genes: The GWAS catalog was downloaded from http://www.genome.gov/admin/gwascatalog.txt in January 2012. Non-disease traits were removed by hand and multiple studies for each disease were combined into unique sets. 'Reported genes' were extracted to provide the list of GWAS genes for each disease.

Drug targets: Drugbank data were downloaded from http://www.drugbank.ca/downloads in January 2012. Drugs for each disease in the GWAS list were identified by searching the 'indication' information for all drugs in Drugbank. Then for each of these drugs, we extract all of the corresponding target genes.

Verified drug targets: Drug targets with the entry "Pharmacological action" labeled as 'Yes' in the Drugbank.

All 4013 GWAS reported genes and 1463 drug targets were mapped to NCBI gene IDs to provide unique identifiers for comparison. For the 88 GWAS diseases with drugs in Drugbank, there are 1914 GWAS reported genes and 821 drug targets. The verified drug target set has 353 genes for 81 diseases. For each disease, we compare the list of GWAS reported genes and drug targets and find the overlap between these two lists.

### Calculating expected overlap between GWAS reported genes and drug targets using a random model

We assume there are 20,000 human genes. For a specific disease, if there are 'm' GWAS reported genes, and there are 'n' drug targets for this disease the expected random overlap between the two gene lists for that disease is n*m/20000. We calculated the expected overlap for each disease and summed these to get the expected total number of overlaps between drug targets and GWAS reported genes for the same disease.

### SNP impact analysis for GWAS genes and drug target genes

1000 genomes VCF data were downloaded from http://www.1000genomes.org/data. The 2010 November data set is used. We extracted all non-synonymous variants from 1000 genomes data based on Refseq annotation downloaded from the UCSC genome browser in Jan 2012, and calculated the allele frequency for each of the non-reference variants by dividing the number of alleles (count 1 for heterozygous and 2 for homozygous) by the number of total possible (2 times the number of people). We found non-synonymous SNPs in the coding regions of 3550 out of the 4013 GWAS reported genes and 1249 out of the 1463 drug targets.

The density of common non-synonymous SNPs in each gene is calculated by dividing the number of non-synonymous SNPs with frequencies > 5% for that gene by the length of that gene's protein sequence provided by the UCSC genome browser http://genome.ucsc.edu/. One splicing form is randomly chosen for each NCBI gene ID.

### Transcript length analysis

The longest transcript for each drug target and GWAS reported gene was picked based on the Refseq annotation downloaded from the UCSC genome browser in Jan 2012.

### Evolutionary analysis for GWAS reported genes and Drug target genes

Ratios of non-synonymous to synonymous substitution rates, dN/dS, for human proteins were downloaded from http://www.h-invitational.jp/evola/download.html in March 2012. The h-inv [34] IDs were converted to NCBI Gene IDs using a conversion map downloaded from http://biodb.jp/download.cgi. dN/dS from Human-Mouse orthologs and Human-Chimpanzee orthologs were selected. Human-Mouse dN/dS are considered to reflect selection over a relatively long time period, and Human-Chimpanzee dN/dS to reflect more recent history.

### Human gene network analysis for GWAS reported genes and drug target genes

The Functional Interaction protein network [21] was downloaded from http://genomebiology.com/content/supplementary/gb-2010-11-5-r53-s3.zip. This un-weighted map consists of 209,988 functional interactions involving 10956 proteins, and covers roughly half of the human coding genome. Gene symbols in this data set were converted to NCBI gene IDs. 1125 out of 1914 GWAS reported genes and 611 out of 821 drug target genes for the 88 diseases and 932 drug targets of all 1463 drug targets were mapped into the network.

The Floyd-Warshall algorithm [35] was used to calculate the shortest path between all gene pairs in the network. The resulting set of inter-node distances serves as a background distribution. For each disease, we extracted the set of all pairwise distances between GWAS genes for that disease, between drug targets genes, and between GWAS genes and drug target genes. For each disease, we also calculated the shortest path from every gene in the network to the nearest GWAS gene for that disease and to the nearest drug target for the disease.

### Machine learning for drug targets

We used a random forest implemented in WEKA [27] to train on the N+3 features to predict known drug targets for a disease from the set of all drug targets. The training sets are unbalanced since the number of drug targets for each disease is very small (median 28) compared to all possible drug targets, 932. We use the MetaCost procedure [36] to deal with the unbalanced training set, which gives more penalty to false negative errors than to false positive errors. We set the cost factor to be the ratio between the number of 'correct' and 'incorrect' drug targets. We set the parameter K, the number of separating features, as the square root of the number of all features and set the parameter I, the number of decision trees in the random forest, as 50. 10 fold cross validation was used to measure the performance for the random forest method for each disease.

## Discussion

This work began with an evaluation of the capability of GWA studies to identify existing drug targets for complex trait disease, based on a comparison of proposed disease mechanism genes in the GWAS catalog and drug targets in Drugbank. To our surprise, only 20 of these 856 drug targets correspond to GWAS identified mechanism genes. Although the point is not emphasized there, a recent study also found a small level of overlap between GWAS disease genes and corresponding drug targets for approved drugs [37] (16 compared with our 20, based on fewer GWAS genes, Table S3 in [37]). Interestingly, that study found that inclusion of targets for drugs at all stages of development boosts the overlap considerably, to 63. Thus it appears that drugs currently being developed are more commonly GWAS genes than those already approved, perhaps because new studies are now selecting targets from GWAS results. Another study has examined the possibility of repurposing based on overlap between OMIM disease genes and drug targets [38], and reports a higher level of overlap.

We investigated two possible reasons why the overlap of GWAS results and drug targets is so low. First, there may be more selection against SNPs with significant impact in drug targets. Studies [5] have shown that GWAS methods typically find high frequency SNPs with modest phenotype effects. On the other hand drug targets have big effect sizes with respect to disease phenotypes. Thus there may be fewer high frequency deleterious SNPs in these genes. Indeed, we do observe this trend for non-synonymous SNPs through analysis of population genomics data from the 1000 genomes project. It is likely that SNPs exerting their influence through other mechanisms (for example, altering the regulation of the expression of genes, changing the splicing pattern, or changing the stability of messenger RNA) also follow the same pattern since selection pressure is independent of impact mechanism. This finding of apparent selection pressure against variants with impact on drug target activity is supported by the observation of similar trends in acceptance of species-specific changes, as measured through dN/dS.

The second possible reason why GWAS genes and drug target overlap is small that we investigated concerns the relative length of GWAS genes versus drug targets. We find that on average GWAS genes are very significantly longer than drug targets, by about a factor of two, and also longer than the set of all genes. These data suggest that mechanisms that are more likely to occur in longer transcripts, such as those involving missense SNPs, play a significant role in complex traits. The data do not rule out other explanations for the length differences, but in any case there is a strong length bias in GWAS genes.

These two factors - selection against common SNPs in drug targets and longer length of GWAS genes - are significant but may not be the only factors contributing to very low drug target/GWAS gene overlap. As discussed earlier, loss of overlap from data errors does not appear large, but incomplete coverage by typical microarrays is a contributing factor [3]. There are some other factors that will contribute. Drugs may act to alleviate symptoms rather than affect the disease itself or they may act in a more global non-specific manner, for example generally suppressing inflammation rather than influencing a specific disease. Also, drugs typically decrease the *in vivo *activity of the protein concerned, whereas altered activity of mechanism genes may affect disease traits through either a decrease or an increase of *in viv*o activity (for example, a SNP may result in up-regulation of expression, contributing to disease risk).

The fact that most existing drug targets are not rediscovered by GWAS does not necessarily imply that few new drug targets will be directly discovered through this technology. For example, many drug targets for inflammatory diseases provide general reduction of inflammation, while its possible that GWAS may lead to much more disease specific targets. What is clear is that the close relationship between drug targets and GWAS reported genes makes the GWAS genes valuable network reference points for finding new drug targets. We have shown that relatively simple machine learning methods are effective at identifying potential drug repurposing opportunities, and one of our initial short-listed repurposing candidates has now been approved for use by the FDA. There is clearly considerable scope for more sophisticated methods, employing a combination of network and pathway information.

The present GWAS technology is only able to detect disease associations involving common SNPs. There are a large number of rare variants in the human exome [39] and as exome sequence and full genome sequence replace DNA microarrays in GWAS studies [40], the role of these is becoming better defined. A deep re-sequencing project for drug target genes has found an abundance of rare functional variants [41] and these are likely to play a role in complex disease. For some diseases, such as hypertension, many candidate genes have been proposed using non-genomic methods [2]. Rare variants in these candidate genes in patients will also be of great interest.

## Abbreviations used

Genome wide association study: GWAS; Single nucleotide variant: SNV; Single nucleotide polymorphism: SNP; Human Gene Mutation Database: HGMD; Support vector machine: SVM; Radial basis function: RBF.

## Competing interests

The authors declare they have no conflict of interests in relation to this SNP-SIG issue article.

## Authors' contributions

CC and JM conceived this work and participated in its design. CC performed all the analyses and machine learning. CC and JM wrote the manuscript. Both authors read and approved the manuscript.

## Supplementary Material

Additional file 1 Overlap between GWAS reported genes and validated drug targets Click here for file

## Declarations

The publication costs for this article were funded by a grant from the National Institutes of Health GM104436.

This article has been published as part of *BMC Genomics *Volume 15 Supplement 4, 2014: SNP-SIG 2013: Identification and annotation of genetic variants in the context of structure, function, and disease. The full contents of the supplement are available online at http://www.biomedcentral.com/bmcgenomics/supplements/15/S4
