# Supplementary material for: GWAS and drug targets
Source: BMC Genomics. 2014 May 20;15(Suppl 4):S5. doi: 10.1186/1471-2164-15-S4-S5 (PMC4083410; doi:10.1186/1471-2164-15-S4-S5)
Supplement: Additional file 1 — Overlap between GWAS reported genes and validated drug targets [file 1471-2164-15-S4-S5-S1.pdf]

Supplementary table 1 Overlap between GWAS reported genes and validated drug targets

Disease Trait	Number of Drugs	Number of GWAS genes	Number of Drug targets	GWAS overlap, same disease	GWAS overlap, all diseases
Acute lymphoblastic leukemia	6	19	5	0	1
Allergic rhinitis	69	11	11	0	0
Alzheimer's disease	5	54	2	0	1
Amyotrophic lateral sclerosis	3	26	2	0	0
Ankylosing spondylitis	39	17	6	0	1
Arthritis	168	7	49	0	1
Asthma	102	43	25	1	2
Atopic dermatitis	12	8	3	0	0
Atrial fibrillation	45	7	14	0	0
Attention deficit hyperactivity disorder	3	81	1	0	3
Autism	3	6	2	0	0
Basal cell carcinoma	6	8	4	0	0
Bipolar disorder/Schizophrenia	93	215	15	0	12
Blood pressure/Hypertension	351	100	71	3	4
Breast cancer	84	42	24	1	4
Celiac disease	3	74	1	0	4
Chronic kidney disease	8	69	6	0	1
Chronic lymphocytic leukemia	14	17	24	0	0
Chronic myeloid leukemia	6	9	7	0	1
Chronic obstructive pulmonary disease	14	18	7	0	2
Colorectal cancer	8	14	4	0	0
Coronary heart disease	6	84	1	0	4
Crohn's disease	7	136	8	0	7
Cystic fibrosis	8	7	7	0	0
Depression/Depressive disorder	45	68	26	0	3
Diabetes	46	205	29	3	11
Duodenal ulcer	8	2	15	0	0

Emphysema	10	5	17	0	0
Endometrial cancer	1	2	1	0	0
Endometriosis	5	4	6	0	0
Epilepsy	18	1	33	0	0
Gallstones	1	1	1	0	0
Gastric cancer	2	3	1	0	2
Glaucoma	24	13	20	0	0
Glioblastoma	2	1	1	0	0
Heart failure	51	16	40	0	1
HIV/AIDS	54	62	24	1	3
Hodgkin's lymphoma	8	7	18	0	0
Hypertriglyceridemia	2	5	4	0	1
Hypothyroidism	5	43	5	0	3
Inflammatory bowel disease	2	18	5	0	0
Kawasaki disease	1	20	11	1	1
Malaria	17	3	8	0	1
Male infertility	6	5	3	0	1
Melanoma	9	20	5	0	2
Menopause age	9	23	6	0	0
Migraine	20	7	25	0	1
Multiple myeloma	7	3	9	0	0
Multiple sclerosis	10	123	22	0	5
Myocardial infarction	29	14	23	0	0
Narcolepsy	2	4	1	0	0
Nephropathy/Nephrotic syndrome	20	26	27	0	0
Neuroblastoma	2	2	5	0	0
Non-small cell lung cancer	5	7	8	0	0
Obesity	4	40	9	0	3
Osteoarthritis	26	3	11	0	0
Osteoporosis	13	10	4	0	0
Ovarian cancer	5	10	2	0	0
Paget's disease	4	9	1	0	0
Pancreatic cancer	2	29	4	0	0
Panic disorder	6	10	3	0	1
Parkinson's disease	20	62	31	0	0
Polycystic ovary syndrome	2	7	2	0	2
Prostate cancer	14	94	12	0	4
Psoriasis/Psoriatic	19	30	17	0	0

arthritis					
Refractive error	1	4	1	0	0
chronic kidney disease	4	4	6	0	0
Restless legs syndrome	2	6	2	0	0
Rheumatoid arthritis	46	67	38	1	3
Sleepiness	1	2	1	0	1
Stevens-Johnson syndrome/toxic epidermal necrolysis	1	12	1	0	0
Stroke	8	4	5	0	1
Tardive dyskinesia	3	1	2	0	0
Testicular cancer	4	7	5	0	0
Thyroid cancer	2	5	3	0	0
Tuberculosis	12	5	12	0	0
Type 1 diabetes	8	74	5	0	3
Type 2 diabetes	28	91	19	2	7
Ulcerative colitis	5	95	9	1	5
Uterine fibroids	1	7	1	0	0
Venous thromboembolism	1	7	1	0	1
Averages	21.3	30.6	11.2	0.17	1.4
